# Effects of substituting alfalfa silage with whole plant quinoa silage on rumen fermentation characteristics and rumen microbial community of sheep *in vitro*

**DOI:** 10.3389/fvets.2025.1565497

**Published:** 2025-04-09

**Authors:** Jie Bai, Lijuan Tang, Mao Liu, Ting Jiao, Guiqin Zhao

**Affiliations:** ^1^Key Laboratory for Grassland Ecosystem of Ministry of Education, College of Pratacultural Science, Gansu Agricultural University, Lanzhou, China; ^2^Provincial R and D Institute of Ruminants in Gansu, Lanzhou, China

**Keywords:** sheep, rumen microbiota, rumen fermentation characteristics, alfalfa silage, quinoa silage

## Abstract

This study investigated the effect of different ratios of quinoa-to-alfalfa silage on the fermentation parameters, methane production, and rumen microbial community composition during *in vitro* fermentation trials. The objective was to evaluate the potential of quinoa as a viable silage material. Five treatment groups were set up with varying quinoa proportions of 0, 30, 50, 70, and 100%, and stored 60 days. The results showed that increasing the quinoa proportion in the alfalfa-quinoa mixed silage resulted in a decrease in concentrations of propionate, isobutyrate, isovalerate, and the methane (CH_4_) fraction of total gas emissions (*p* < 0.05). Conversely, dry matter digestibility, total volatile fatty acid (TVFA) concentration, acetate concentration, acetate to propionate ratio, butyrate concentration, cumulative CH_4_ emissions, and total gas production increased (*p* < 0.05). At the phylum level, the relative abundance of Spirochaetota decreased linearly (*p* < 0.05), while Verrucomicrobiota increased (*p* < 0.05). At the genus level, the relative abundance of *CAG 873*, *Prevotella*, *Acinetobacter*, *Treponema D*, *RUG11690*, and *Ruminococcus E* decreased linearly (*p* < 0.05), whereas the relative abundance of *Bact 11*, *Limimorpha*, *F23 D06*, *Advenella*, and *unclassified bacteria* increased linearly (*p* < 0.05). In summary, the inclusion of quinoa in alfalfa silage alters the fiber structure of the feed and significantly affects its nutritional composition, *in vitro* fermentation parameters, methane production, and microbial community composition. These findings offer valuable insights for optimizing ruminant feed.

## 1 Introduction

Alfalfa (*Medicago sativa L.*), known for its high-protein forage, is a valuable forage for ruminants such as dairy cows, sheep. However, its availability in China is limited due to inconsistent quality and relatively small scale of cultivation. This has led to a shortage of alfalfa to meet the increasing demands of livestock (1). To address this issue, it is crucial to expand high-quality forage resources. One potential solution is the cultivation of highly adaptable crops and forage species on marginal, saline-alkali, abandoned, and idle agricultural lands in China. This strategy could increase the overall forage production area and help bridge the supply–demand gap for herbivorous livestock feed.

Quinoa is a highly adaptable crop that can thrive in various agro-ecological environments, demonstrating notable resistance to frost, salinity, and drought (2, 3). It is also capable of growing on marginal soils (4, 5). Traditionally cultivated in high-altitude regions above 3,000 m, where annual precipitation averages around 300 mm, quinoa typically reaches a height of approximately 1.0 m. In China, quinoa is grown at elevations ranging from 700 to 3,000 m, with most cultivation occurring below 2,000 m. In these areas, characterized by higher temperatures and increased precipitation, quinoa plants can grow taller, often exceeding 1.8 m and occasionally reaching up to 3 m. This results in larger biomass compared to its native growing regions (6–8). The quinoa plant is rich in essential amino acids, including lysine, threonine, and methionine (9–11), as well as high concentrations of phenolics and flavonoids (12, 13), which contribute to its antimicrobial and antioxidant properties (14–17). These attributes make quinoa a promising alternative to traditional animal feed.

Recently, quinoa has gained global attention as a potential forage crop, due to the high nutritional value of its whole plant for livestock (18). The vegetative portion of quinoa is particularly rich in protein and accumulates significantly more biomass than the grain itself (19). In several developed livestock-producing regions, the whole plant quinoa is being used as animal feed (20). Asher et al. (21) analyzed the nutritional profile of both whole plant quinoa hay and the residual straw left after seed harvesting. Their findings indicated crude protein levels of 19.9 and 10.6%, respectively, with *in vitro* DM digestibility rates of 75.8 and 54.2%. In a similar study, Peiretti et al. (22) used *in vitro* methods to assess the nutritional properties of quinoa, reporting DM digestibility between 710 and 900 g/kg DM, and NDF digestibility ranging from 430 to 840 g/kg NDF. Kakabouki et al. (23) evaluated the potential of the whole plant quinoa as a ruminant feed, suggesting that quinoa could serve as a viable alternative to local legume forages in the Mediterranean region. While these results are promising, research on the effects of whole plant quinoa on rumen microbiota remains limited.

Although quinoa is often praised for its nutritional benefits, comprehensive data on the full nutritional value of the whole plant quinoa as feed for ruminants is lacking. Additionally, ensiling is a key technique for preserving moist forage, ensuring a continuous feed supply for livestock throughout the year (24). However, the impact of whole plant quinoa silage on rumen fermentation patterns and microbial populations has yet to be thoroughly explored. Therefore, this study aimed to investigate the chemical composition and in sacco degradability of whole-plant quinoa silage, as well as assess the *in vitro* ruminal fermentation kinetics of diets in which quinoa replaced alfalfa in five ratios.

## 2 Materials and methods

### 2.1 Harvest of quinoa and alfalfa, silage preparation, and chemical composition analysis

Both alfalfa and quinoa were cultivated in experimental fields located in Jingchuan, Gansu, China. The alfalfa was harvested at the onset of its flowering stage, while the quinoa was collected during its early blooming phase. Samples were obtained from three experimental plots (approximately 4 m^2^ each) on July 20, 2022, and chopped into 2–3 cm pieces. Alfalfa and quinoa samples were randomly selected and blended in various wet weight ratios: 1:0 (Q0, Control), 0.70:0.30 (Q30), 0.50:0.50 (Q50), 0.30:0.70 (Q70), and 0:1 (Q100). A 500 g portion of each blend was vacuum-sealed in plastic bags (25 cm × 35 cm). Three replicate bags were prepared for each treatment, and the silage was allowed to ferment for 60 days at an ambient temperature.

After 60 days of ensiling, a 150 g sample was dried for 72 h in a forced-oven to quantify the dry matter (DM) content, then ground using a 1 mm sieve. Neutral detergent fiber (aNDF) and acid detergent fiber (ADF) levels were determined according to the Association of Official Analytical Chemists (AOAC) protocols (25, 26). Crude protein (CP) was calculated using the formula TN × 6.25. Water-soluble carbohydrates (WSC) were measured based on the procedure described by Murphy (27).

### 2.2 *In vitro* fermentation trial

Rumen fluid and buffered inoculum were prepared according the method described by Menke and Steingass (28). Three sheep (59.28 ± 4.03 kg), each fitted with a permanent rumen cannula, were fed a nutritionally balanced diet consisting of alfalfa pellets and concentrate in a 70:30 (DM basis). The diet was offered twice daily at 08:00 and 18:00 h. Rumen fluid was collected from each sheep before the morning feeding, filtered through four layers of medical gauze, and then pooled in equal volumes in a pre-warmed (39°C) thermos flask. The mixture was transported to the laboratory within 30 min. Buffered inoculum was prepared by blending the rumen fluid with a pre-warmed (39°C) buffer solution in a 1:2 (v/v) ratio, under a continuous flow of carbon dioxide.

*In vitro* incubations were conducted following the method described by Menke et al. (29). Approximately 400 mg of silage was placed in a filter bag (pore size: 25 μm) and securely sealed. A total of 44 filter bags were prepared: two bags for each sample and four blank bags without any sample. Prior to incubation, the bags were pre-warmed to 39°C. Each bag was then submerged in a 100 mL glass syringe containing 40 mL of buffered inoculum. The syringes were incubated for 36 h in a water bath shaker, gently agitated at 60 rpm, and maintained at 39°C. Gas volume was measured manually at 36 h. At each sampling interval, 5 mL gas samples were collected using a vacuum blood collection tube and stored at 4°C for subsequent CH_4_ analysis.

### 2.3 *In vitro* ruminal fermentation profiles and methane analysis

After 36 h of incubation, the bags were carefully removed from the syringes, rinsed with tap water, and freeze-dried for 72 h. Once dried, the samples were weighed to determine *in vitro* dry matter disappearance (IVDMD). Concurrently, the incubated liquid was transferred into 50 mL centrifuge tubes, where the pH was measured before the liquid was split into two separate subsamples. The first subsample was used to measure NH_3_-N (30) and volatile fatty acids (VFA), including acetate, propionate, butyrate, isobutyrate, valerate, and isovalerate, following centrifugation at 10,000 × g for 5 min at 4°C, as described by Chen et al. (31). The second subsample was stored in a 10 mL centrifuge tube for DNA extraction. Methane (CH_4_) concentration was then quantified using the method outlined by Chen et al. (31).

### 2.4 High throughput sequencing and analysis

Total microbial DNA was extracted from rumen fluid using the E.Z.N.A.® Stool DNA Kit (Omega BioTEK, Norcross, GA, USA) following the manufacturer’s protocol. The quality and purity of the extracted DNA were assessed using 1% agarose gel electrophoresis and quantified with a spectrophotometer (NanoDrop 2000C, Thermo Scientific, USA). DNA samples meeting the quality standards were stored at −20°C for subsequent analysis. The bacterial 16S rRNA gene V3-V4 region was amplified using primers 338F (5’-ACTCCTACGGGAGGCAGCAG-3′) and 806R (5’-GGACTACHVGGGTWTCTAAT-3′). PCR products were analyzed by 1% agarose gel electrophoresis and purified with the Agencourt® AMPure® XP nucleic acid kit. The purified PCR products were then used for library construction and sequenced using the Illumina Miseq PE300 platform.

Raw sequences generated from sequencing were processed and filtered using QIIME1 (v1.8.0), Pear (v0.9.6), and Vsearch (v2.7.1) software to obtain high-quality data. These sequences were then aligned with the Gold Database to verify their suitability for further analysis. Subsequent analyses were performed using Vsearch (v2.7.1), where sequences were clustered into OTUs based on a similarity threshold of >97%. The RDP Classifier algorithm was used to assign taxonomy to each OTU by aligning them with the Silva128 database. Finally, the OTU data were normalized using the minimum rarefaction method, and microbial alpha diversity indices were calculated.

### 2.5 Statistical analysis

Data on the chemical profile of silage, CH_4_ emissions, and *in vitro* rumen fermentation parameters and bacterial compositions were analyzed using the GLM procedure in SAS software. The model used was:


$$Y_{i}=\mu +t_{i}+\varepsilon_{i}$$

where Y_i_ represents the observed value, μ is the mean, t_i_ denotes the treatment effect, and ε_i_ refers to the residual error. Means were compared using Duncan’s multiple comparison test, with significance set at *p* < 0.05. The relationships between bacterial communities and correlations between *in vitro* fermentation parameters and gas emissions were examined using R statistical software.

## 3 Results

### 3.1 Chemical profile of ensiled alfalfa, quinoa, and their mixture silage

The CP content of the silage decreased linearly with increasing quinoa incorporation in the mixture (Table 1, *p* < 0.05). In contrast, the contents of NDF and ADF increased linearly (*p* < 0.05) as quinoa proportion in the mixture rose.

**Table 1:** Chemical composition of silage prepared with alfalfa, quinoa, and their mixture.

Item^1^	Treatment^2^					Significance^4^			
	Q0	Q30	Q50	Q70	Q100	SEM^3^	T	L	Q
DM (%)	30.82	29.03	28.82	29.96	30.08	0.4552	0.6926	0.871	0.420
CP (%)	17.62^a^	17.63^a^	16.91^a^	13.42^b^	11.03^c^	0.7350	<0.0001	<0.0001	<0.0001
NDF (%)	33.65^c^	35.45^bc^	37.80^b^	37.65^b^	42.76^a^	0.8759	0.0002	<0.0001	<0.0001
ADF (%)	21.39^d^	23.63^bc^	22.85^cd^	25.03^b^	27.52^a^	0.6008	0.0003	<0.0001	<0.0001
WSC (%)	4.04	4.00	4.42	4.50	4.87	0.1799	0.5862	0.089	0.236

^1^DM, dry matter; CP, crude protein; NDF, neutral detergent fiber; ADF, acid detergent fiber; WSC, water soluble carbohydrates. ^2^Alfalfa and quinoa were combined in proportions of 1:0 (Q0), 0.70:0.30 (Q30), 0.5:0.5 (Q50), 0.30:0.70 (Q70) and 0:1 (Q100). ^3^SEM, standard error of the mean. ^a–e^means with different superscript letters in the same row differ (*p* < 0.05). ^4^T, treatment; L, linear; Q, quadratic.

### 3.2 *In vitro* rumen fermentation characteristics and methane concentration of alfalfa, quinoa, and their mixture silage

The rumen pH decreased significantly when quinoa supplementation increased to 50% (Table 2, *p* < 0.05). IVDMD, acetate, acetate to propionate ratio and TVFA increased with the increase of quinoa addition in the mix silage, while propionate and decreased. In addition, there was on difference in acetate, propionate, acetate to propionate ratio, isobutyrate, valerate and isovalerate between Q50 and Q70. It was interesting that isobutyrate, valerate and isovalerate of Q50 and Q70 was the same as that of Q0, but significantly higher than that of Q30 and Q100. Cumulative total gas and CH_4_ emissions increased with the quinoa addition increased, while the CH_4_/total gas ratio decreased.

**Table 2:** *In vitro* rumen fermentation parameters and gas emission of silage prepared with alfalfa, quinoa, and their mixture.

Item^1^	Treatment^2^					Significance^4^			
	Q0	Q30	Q50	Q70	Q100	SEM^3^	T	L	Q
pH	6.87^a^	6.85^a^	6.77^b^	6.75^b^	6.70^b^	0.0176	0.0007	<0.0001	<0.0001
NH_3_-N (mg/100 mL)	12.60^b^	12.37^b^	13.22^ab^	16.29^a^	8.22^c^	0.7309	0.0020	0.362	0.047
IVDMD (g/kg DM)	53.92^e^	58.01^d^	61.23^c^	65.64^b^	75.41^a^	1.7327	<0.0001	<0.0001	<0.0001
TVFA (mmol/L)	26.40^c^	28.44^b^	29.94^b^	32.98^a^	34.43^a^	0.7095	<0.0001	<0.0001	<0.0001
Acetate (%)	52.60^d^	59.92^c^	62.40^bc^	63.80^b^	71.40^a^	1.4420	<0.0001	<0.0001	<0.0001
Propionate (%)	30.51^a^	24.74^b^	18.92^c^	17.74^c^	13.50^d^	1.4186	<0.0001	<0.0001	<0.0001
Acetate: propionate	1.74^d^	2.45^c^	3.31^b^	3.62^b^	5.30^a^	0.2859	<0.0001	<0.0001	<0.0001
Butyrate (%)	9.51	10.04	11.85	11.01	11.49	0.3197	0.0888	0.025	0.046
Isobutyrate (%)	1.62^a^	1.11^bc^	1.43^ab^	1.56^a^	0.83^c^	0.0801	0.0004	0.040	0.078
Valerate (%)	2.43^a^	1.86^b^	2.44^a^	2.65^a^	1.38^c^	0.1186	<0.0001	0.125	0.053
Isovalerate (%)	3.31^a^	2.30^b^	2.93^ab^	3.23^a^	1.38^c^	0.1865	<0.0001	0.021	0.711
Cumulative total gas emission (mL/g)	36.86^e^	42.74^d^	47.89^c^	52.65^b^	67.86^a^	2.4598	<0.0001	<0.0001	<0.0001
Cumulative CH_4_ emission (mL/g)	3.65^b^	3.89^ab^	4.16^a^	3.94^ab^	4.22^a^	0.0616	0.0096	0.004	0.010
CH_4_/total gas emission (mL/100 mL)	9.92^a^	9.12^b^	8.70^b^	7.48^c^	6.21^d^	0.3040	<0.0001	<0.0001	<0.0001

^1^NH_3_-N, ammonium nitrogen; DM, dry matter; IVDMD, *in vitro* DM digestibility; TVFA, total volatile fatty acids; CH_4_, methane. ^2^Alfalfa and quinoa were combined in proportions of 1:0 (Q0), 0.70:0.30 (Q30), 0.5:0.5 (Q50), 0.30:0.70 (Q70) and 0:1 (Q100). ^3^SEM, standard error of the mean. ^a–e^means with different superscript letters in the same row differ (*p* < 0.05). ^4^T, treatment; L, linear; Q, quadratic.

### 3.3 *In vitro* ruminal microbiota of alfalfa, quinoa, and their mixture silage

**Figure 1:**
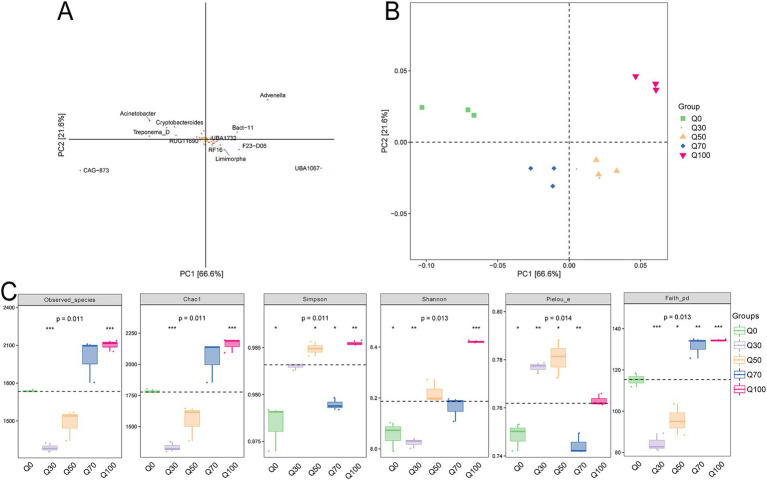
Bacterial diversity. **(A,B)** Species loadings and two-dimensional ordination of samples from PCA. A represents each point as a species (default classification is by genus). The horizontal and vertical coordinates reflect the relative contribution of each species to the variation observed in the samples along the two principal components. The percentages in parentheses on the axes indicate the proportion of compositional differences in species abundance relative to the total variation in each dimension across all samples. The default settings for the two axes ensure that the ratio of the physical length of the axes corresponds to the ratio of their respective variances. This means that the contribution of a species to the compositional differences between sample groups is proportional to the sum of its distances from the axes, with contributions indicated by a color gradient from yellow (smallest) to red (largest). **(B)** Each point in the graph represents a sample, with different colors indicating different sample groups. **(C)** Grouped boxplots of the Alpha diversity index.

According to the PCA loading plot (Figure 1A), Advenella, *CAG 873*, and *UBA1067* contributed significantly to the PCA, suggesting they might be key species responsible for the differences in microbial community composition. The PCA plot depicting the overall rumen bacterial structure (Figure 1B) showed distinct clustering of bacterial community compositions, with Q0 in the first quadrant, Q100 in the second quadrant, Q70 in the third quadrant, and Q30 and Q50 in the fourth quadrant. Except for Q0, the richness of the rumen bacterial community, as measured by the Observed_species and Chao1 indices, increased with the level of quinoa supplementation. Diversity, assessed by the Shannon index, was the highest in the Q100 treatment, followed by Q50 and Q70, while the lowest diversity was observed in the Q0 and Q30 treatments (Figure 1C).

**Figure 2:**
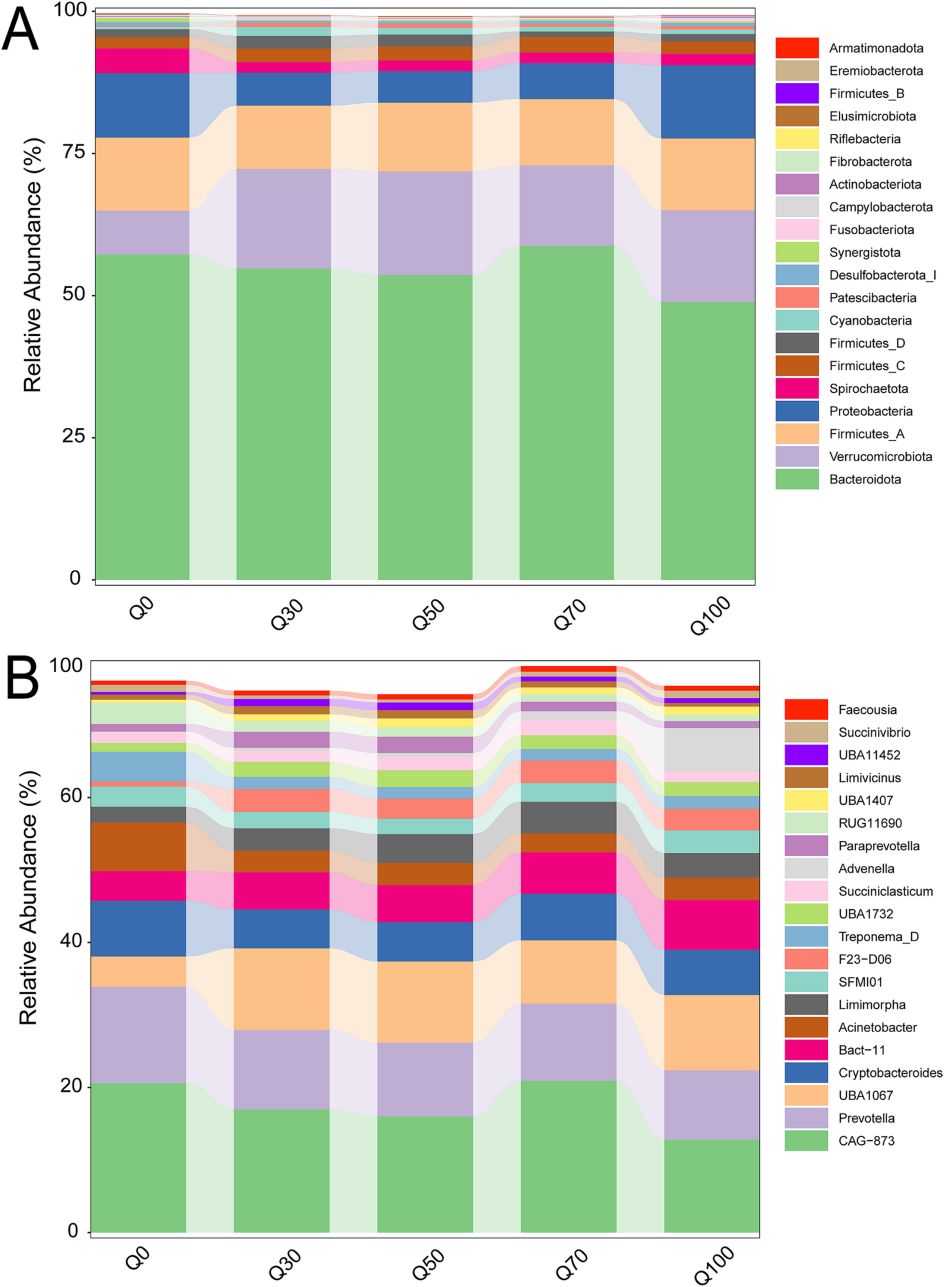
Comparison of rumen bacterial phyla **(A)** and genera **(B)** abundance across different groups.

The bacterial composition structures were shown in Figure 2. Bacteroidetes and Firmicutes were the dominant phyla (Figure 2A), while *Prevotella* and *CAG 873* were the predominant genera (Figure 2B). At the phylum level, the relative abundance of Spirochaetota exhibited a slight linear decrease (Supplementary Table S1, *p* < 0.05), whereas the relative abundance of Verrucomicrobiota showed a linear increase (*p* < 0.05) as the quinoa proportion increased. At the genus level, the relative abundance of *CAG 873*, *Prevotella*, *Acinetobacter*, *Treponema D*, *RUG11690*, and *Ruminococcus E* also displayed a slight linear decrease (Supplementary Table S2, *p* < 0.05), while the relative abundance of *Bact 11*, *Limimorpha*, *F23 D06*, *Advenella*, and *unclassified Bacteria* exhibited a linear increase (*p* < 0.05) with increasing quinoa proportion.

**Figure 3:**
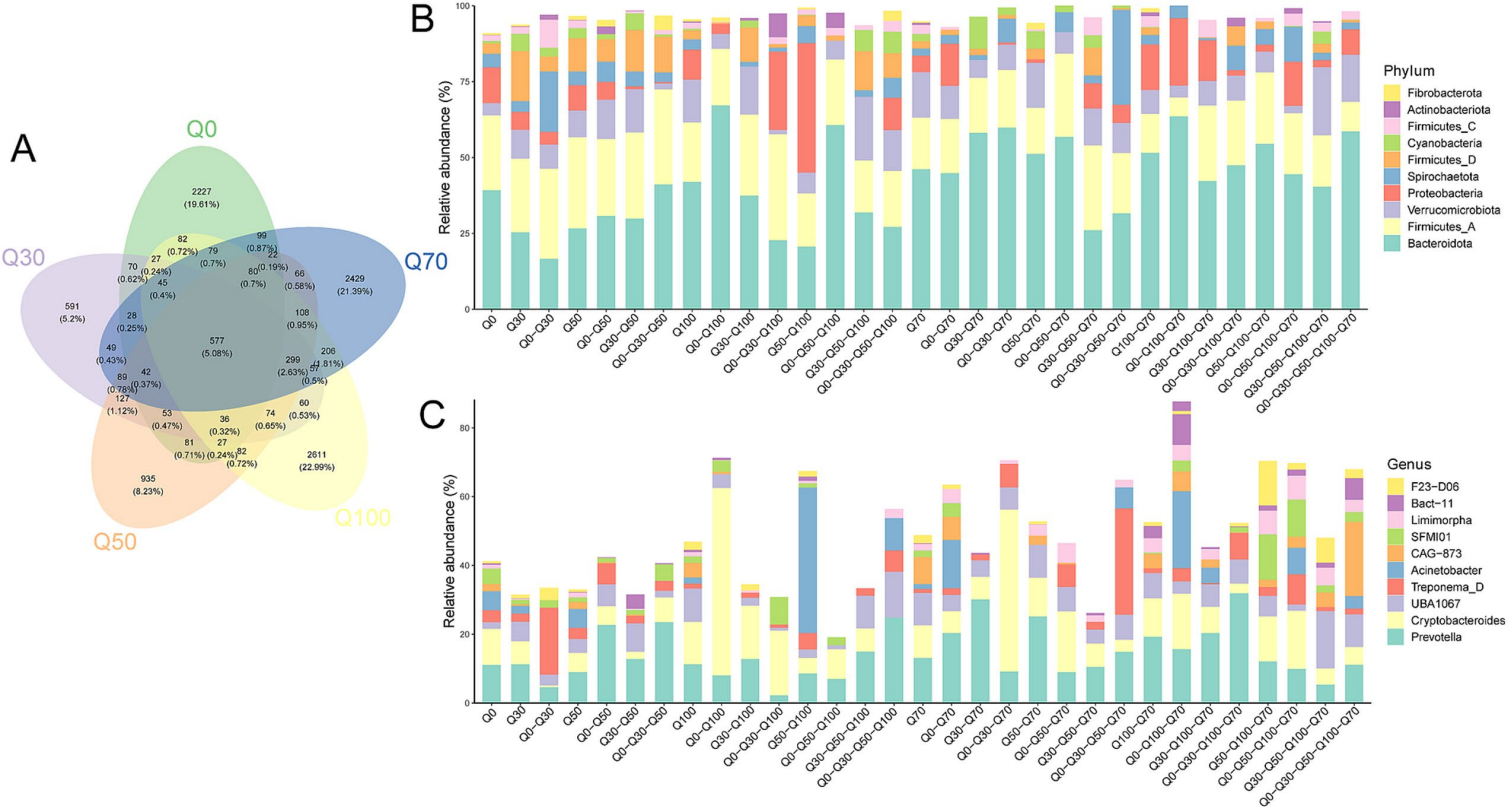
OTU Venn diagram of bacterial communities among treatments. **(A)** Venn diagram of Operational Taxonomic Units (OTUs) across treatments. Each ellipse represents a different treatment, and the overlapping areas indicate shared OTUs between treatments. The numbers in each section correspond to the number of OTUs present in that specific region. **(B,C)** Histograms of OTU abundance in different regions of the Venn diagram. The horizontal axis represents the OTUs located in various regions, while the vertical axis shows the percentage of sequence abundance from different phyla **(B)** and genera **(C)**.

According to the Venn diagram (Figure 3A), the number of unique OTUs in each treatment group were as follows: 2227 for Q0, 591 for Q30, 935 for Q50, 2,429 for Q70, and 2,611 for Q100. The total number of OTUs across all five groups was 577. To further investigate the bacterial composition of each group, the OTUs from each treatment were annotated at both the phylum and genus levels, with the results presented in bar charts. At the phylum level (Figure 3B), the relative abundance of unique bacterial communities in Q0, ranked from highest to lowest, were Bacteroidota, Firmicutes A, and Proteobacteria. In Q30, the order was Bacteroidota, Firmicutes A, Firmicutes D, Verrucomicrobiota, and Cyanobacteria; in Q50, it was Firmicutes A, Bacteroidota, Firmicutes D, Proteobacteria, and Verrucomicrobiota; in Q70, Bacteroidota, Verrucomicrobiota, Firmicutes A, and Proteobacteria dominated; and in Q100, the order was similar to Q70 with Bacteroidota, Verrucomicrobiota, Firmicutes A, and Proteobacteria. At the genus level (Figure 3C), the relative abundance of unique bacterial communities in Q0, ranked from highest to lowest, were *Prevotella*, *Cryptobacteroides*, *Acinetobacter*, *SFMI01*, and *Treponema D*. In Q30, the order was *Prevotella*, *Cryptobacteroides*, and *UBA1067*; in Q50, it was *Prevotella*, *Acinetobacter*, *Cryptobacteroides*, and *UBA1067*; in Q70, the order was *Prevotella*, *UBA1067*, *Cryptobacteroides*, and *CAG 873*; and in Q100, it was *Cryptobacteroides*, *Prevotella*, *UBA1067*, and *CAG 873*.

**Figure 4:**
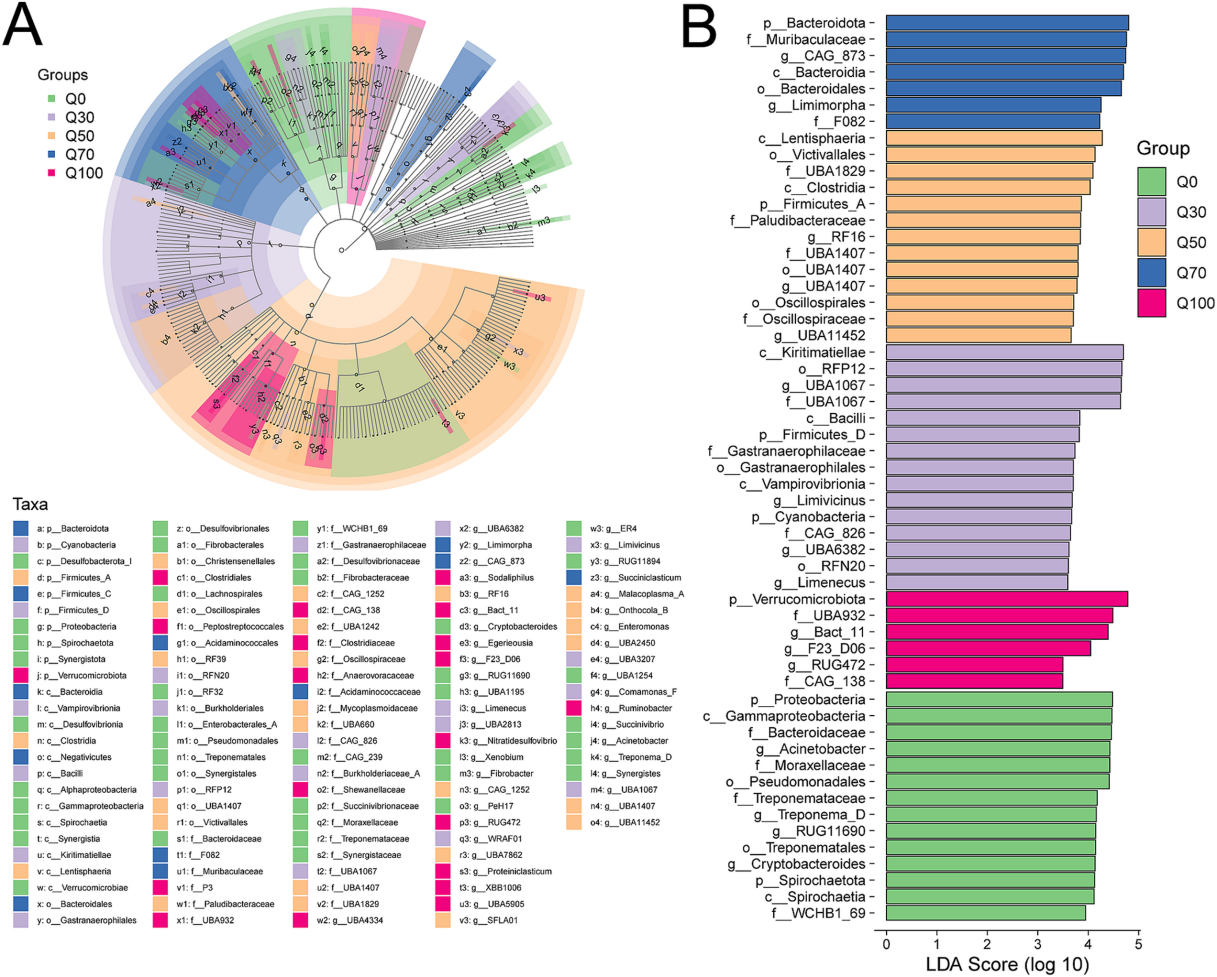
Microbial community differences in different groups. **(A)** LEfSe cladogram comparing microbial communities across different groups. **(B)** Histogram of LDA scores for each taxon, ranging from phylum to genus.

LEfSe was employed to identify variations in bacterial taxa composition. A representative cladogram depicting the predominant microbiome structure is shown in Figure 4A, highlighting the most significant taxonomic differences among the groups. The analysis revealed that fourteen clads were enriched in the Q0 group, fifteen clads in the Q30 group, thirteen clads in the Q50 group, seven clads in the Q70 group, and six clads in the Q100 group. Figure 4B illustrates the distinct bacterial taxa in the five groups. When comparing microbial communities across the groups, the most differentially abundant bacterial genera in Q0 were *Acinetobacter*, *Treponema D*, *RUG11690*, and *Cryptobacteroides*. In Q30, *UBA1067*, *Limivicinus*, *UBA6382*, and *Limenecus* were more prevalent. In Q50, RF16, *UBA11407*, and *UBA111452* were prevalent. In Q70, *CAG 873* and *Limimorpha* were more abundant, and in Q100, *Bact 11*, *F23 D06*, and *RUG472* showed higher relative abundance.

### 3.4 Correlations between rumen bacterial genus and fermentation parameters

**Figure 5:**
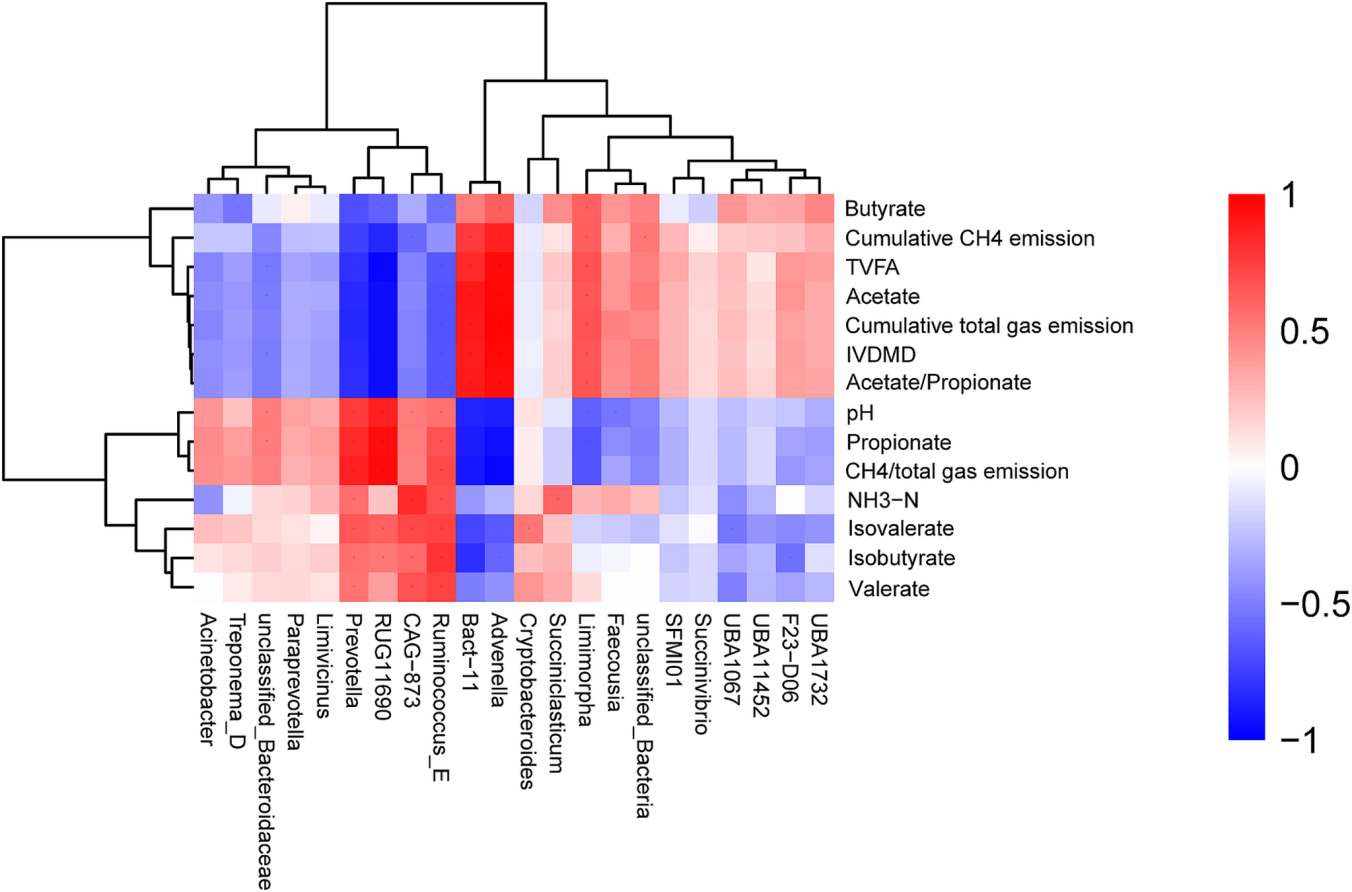
Correlation and clustering analysis between IVDMD, cumulative CH_4_ emissions, cumulative total gas emissions, CH_4_/total gas emission ratio, ruminal fermentation parameters, and bacterial genera.

Cumulative CH_4_ emission, cumulative total gas emission, TVFA, acetate, IVDMD, and acetate to propionate ratio were positively correlated with the relative abundances of the genera *Advenella* and *Bact 11*, while showing negative correlations with the abundances of *Ruminococcus E*, *Prevotella*, and *Acinetobacter* (Figure 5). The values of pH, propionate, and CH_4_/total gas emission were positively correlated with *Prevotella*, *Ruminococcus E*, *Acinetobacter*, and *RUG11690*, but negatively correlated with *Advenella* and *Bact 11* abundances. The NH_3_-N concentration was positively correlated with *Succiniclasticum*, *Ruminococcus E*, and *Prevotella*, and negatively associated with *Acinetobacter* abundance. The isovalerate concentration showed a positive correlation with *Cryptobacteroides* and a negative association with *Advenella*. Similarly, the isobutyrate concentration was positively associated with *Ruminococcus E* and *Prevotella*, but negatively correlated with *Bact 11*. The valerate concentration was positively associated with the relative abundances of the genera *Ruminococcus E* and *CAG 873*, and negatively correlated with the abundance of *UBA1067*.

### 3.5 Tax4Fun gene function estimation

**Figure 6:**
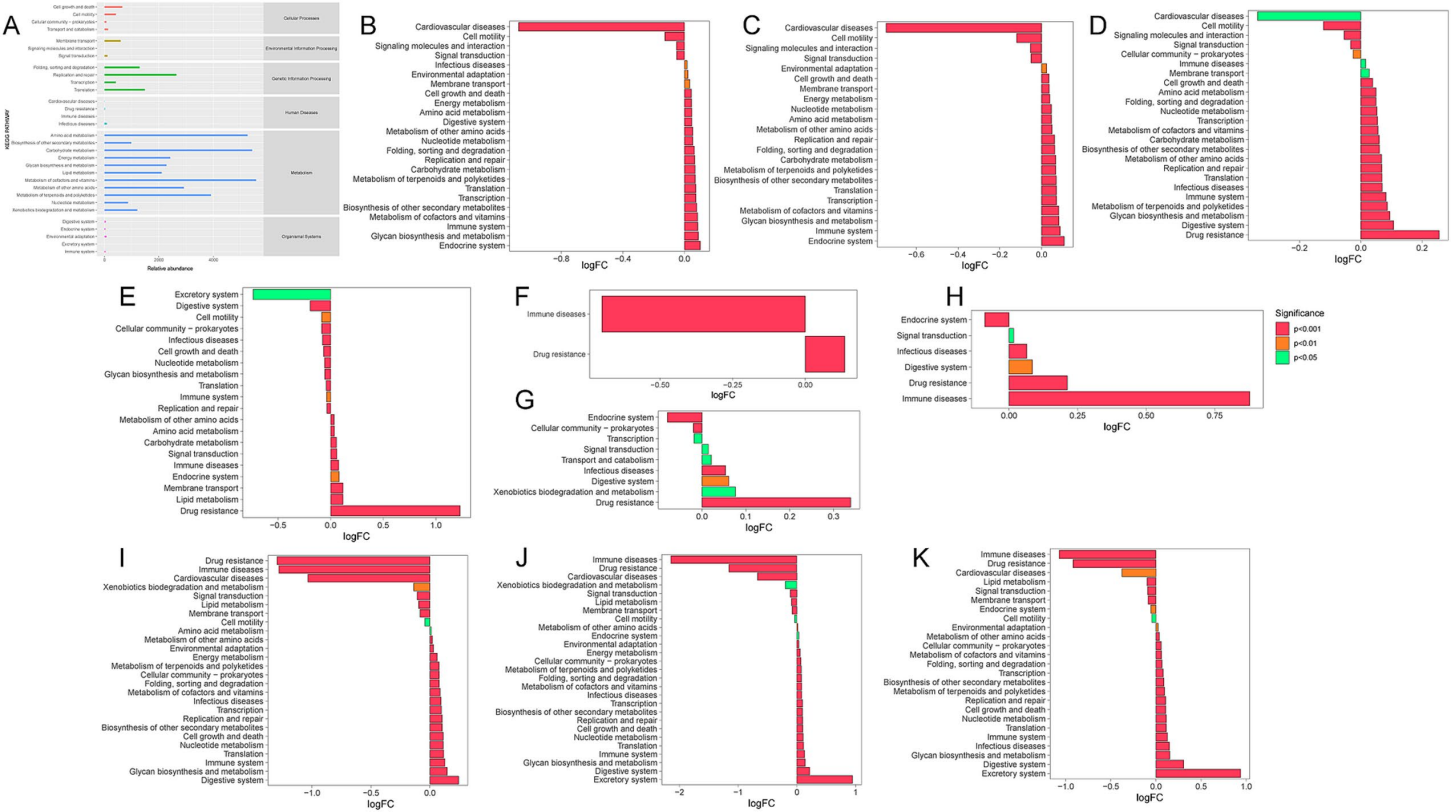
KEGG metabolic pathway maps. **(A)** Predicted KEGG secondary functional pathway abundance maps. **(B–J)** KEGG metabolic pathways differing between two groups. Positive logFC values on the horizontal axis indicate up-regulation in group B relative to group A (where group A refers to the name preceding “vs.” and group B refers to the name following “vs.”). Negative values indicate down-regulation. **(B)** Q0 vs. Q30; **(C)** Q0 vs. Q50; **(D)** Q0 vs. Q70; **(E)** Q0 vs. Q100; **(F)** Q30 vs. Q50; **(G)** Q30 vs. Q70; **(H)** Q50 vs. Q70; **(I)** Q100 vs. Q50, **(J)** Q100 vs. Q70; **(K)** Q100 vs. Q30.

Tax4Fun was used to predict the functional characteristics of the rumen microbiota. The KEGG biological metabolic pathway analysis (Figure 6A) categorized the metabolic pathways into six main groups: metabolism, genetic information processing, environmental information processing, cellular processes, organismal systems, and human diseases. The predominant metabolic pathways in the rumen microbiota included those related to metabolism (particularly cofactors and vitamins, carbohydrates, amino acids, and terpenoids and polyketides) and genetic information processing (specifically replication and repair). MetagenomeSeq was employed to identify significant differences in metabolic pathways across the groups (Figures 6B–K). As quinoa inclusion increased, several microbial functions showed significant alterations. These functions encompassed environmental adaptation, cell growth and death, membrane transport, energy metabolism, nucleotide metabolism, amino acid metabolism, metabolism of other amino acids, replication and repair, protein folding, sorting and degradation, carbohydrate metabolism, metabolism of terpenoids and polyketides, biosynthesis of secondary metabolites, translation, transcription, metabolism of cofactors and vitamins, glycan biosynthesis, immune system functions, and endocrine system processes. All of these functions exhibited a significant increase with higher quinoa supplementation.

## 4 Discussion

### 4.1 Ensiling characteristics of ensiled alfalfa, quinoa, and their mixture

The experimental results indicated that increasing the proportion of quinoa in the mixed silage leads to significant changes in its nutritional composition. Specifically, the CP content of the silage decreased linearly as the proportion of quinoa increased, likely due to quinoa’s inherently lower protein content (19). When the quinoa proportion exceeded 70%, the CP content was significantly lower compared to pure alfalfa silage, suggesting that the addition of quinoa dilutes the protein concentration in the silage. Simultaneously, the contents of NDF and ADF increased linearly with higher quinoa inclusion. This trend is consistent with the nutritional characteristics of quinoa, which tends to have higher fiber levels compared to alfalfa (8). These findings emphasize the need to carefully balance the inclusion of quinoa in silage mixtures to maintain desired protein and fiber levels for optimal nutritional value.

### 4.2 *In vitro* rumen fermentation profiles and gas emission in ensiled alfalfa, quinoa, and their mixture

The experimental results indicated that as the proportion of quinoa in the silage increased, there was a decrease in ruminal pH, as well as in the concentrations of propionate, isobutyrate, isovalerate, and the proportion of CH_4_ in the total gas produced. In contrast, IVDMD, TVFA concentration, acetate concentration, the acetate to propionate ratio, butyrate concentration, cumulative CH_4_ emissions, and cumulative total gas emissions all increased. These findings suggest that the addition of quinoa significantly influences ruminal fermentation and gas emissions, likely by altering the metabolic activity of the ruminal microbial community and the composition of fermentation products. The observed decrease in ruminal pH may inhibit the synthesis of propionate, isobutyrate, and isovalerate (10). Compared to alfalfa, quinoa contains higher fiber content and fewer fermentable sugars, which likely contribute to the lower concentrations of propionate, isobutyrate, and isovalerate in the fermentation products (9).

Conversely, as the proportion of quinoa increased, the TVFA concentration exhibited a significant linear increase. This trend is likely linked to the enhanced production of acetate and butyrate. Acetate, as a primary energy source for ruminants, is typically associated with fiber fermentation (32). Given that quinoa contains higher fiber content and exhibits greater fiber conversion efficiency, it may promote increased acetate production during fermentation (18). The observed rise in butyrate concentration further supports this hypothesis, as butyrate is a major product of fiber carbohydrate fermentation (33). Thus, quinoa’s higher fiber content likely promotes butyrate production. Moreover, the increase in the acetate to propionate ratio suggested a significant shift in the relative production of these two acids. This change indicated that the addition of quinoa might alter the metabolic pathways of the ruminal microbial community, ultimately affecting the production ratio of acetate and propionate (34).

Regarding gas emissions, as the proportion of quinoa increased, the proportion of CH_4_ in total gas emissions significantly decreased. This change was likely associated with quinoa’s higher fiber content and lower fermentable sugar content. Methane production in the rumen was primarily driven by the activity of *Methanobacteriales* (35). The addition of quinoa might inhibit methanogen activity by altering the hydrogen supply in the rumen (36). Compared to alfalfa, quinoa’s coarse fiber might lead to prolonged fermentation, generating more hydrogen, which in turn promoted acetate production (37). Since hydrogen serves as a substrate for methane production, its reduced availability could directly inhibit methane generation (38).

### 4.3 *In vitro* rumen bacterial community in ensiled alfalfa, quinoa, and their mixture

As the proportion of quinoa increased, significant changes occured in the structure of the rumen microbial community, particularly in the relative abundance at both the phylum and genus levels. The experimental results showed that the relative abundance of Spirochaetota decreased linearly, likely due to the increased content of NDF and ADF. Microorganisms in the Spirochaetota phylum are primarily involved in the fermentation and degradation of plant fibers. Quinoa’s higher fiber content might inhibit the activity and proliferation of these microorganisms by altering the degradability of the fibers (39). In contrast, the relative abundance of Verrucomicrobiota increased linearly. This trend might indicate the enhanced adaptability of certain microbial populations to the high-fiber environment introduced by quinoa, or the increased relative dominance of some anaerobic bacteria in this context (40). This shift suggested that quinoa’s fiber composition not only influenced the overall structure of the microbial community but also altered the competitive dynamics among microbial populations in high-fiber environments.

At the genus level, the relative abundance of *CAG 873*, *Prevotella*, *Acinetobacter*, *Treponema D*, *RUG11690*, and *Ruminococcus E* decreased significantly. These genera were involved in fiber degradation, lactic acid fermentation, and carbohydrate breakdown in the rumen (41). As the quinoa proportion increased, changes in fiber composition might reduce the relative abundance of these microbial communities, which were closely linked to cellulose degradation (42). Additionally, quinoa’s higher content of monosaccharides and oligosaccharides might increase competition for *Prevotella*, thereby inhibiting its growth and fermentation activity. In contrast, the relative abundance of *Bact 11*, *Limimorpha*, *F23 D06*, and *Advenella* increased, suggesting that specific components in quinoa create favorable conditions for the growth of these microorganisms. Genera such as *Bact 11* and *Limimorpha* might be particularly well-suited to utilize the soluble sugars or other non-fiber components in quinoa, thereby promoting their proliferation (8).

### 4.4 Relationships between rumen bacterial genera and *in vitro* fermentation parameters, as well as gas emissions

In this study, a significant positive correlation was found between cumulative CH_4_ emissions, cumulative total gas emissions, TVFA, acetate, and IVDMD with the relative abundance of *Advenella* and *Bact 11* genera. This suggested that *Advenella* and *Bact 11* may play a key role in efficient fermentation processes. Previous studies have shown that these bacterial genera are closely linked to efficient fermentation metabolism, particularly exhibiting enhanced activity during cellulose degradation and the synthesis of fermentation products (43). In the rumen of ruminants, an increased relative abundance of *Advenella* and *Bact 11* accelerated the degradation of organic matter and the synthesis of VFAs, thereby enhancing overall fermentation efficiency. This, in turn, leads to increased gas emissions and VFA yields (44, 45). In contrast, the relative abundance of *Ruminococcus E*, *Prevotella*, and *Acinetobacter* showed a negative correlation with these indicators, possibly reflecting their involvement in specific metabolic pathways in the rumen. Specifically, certain bacterial genera might reduce the efficiency of organic matter degradation or interfere with the production of fermentation products through mechanisms such as competitive inhibition, re-utilization of degradation metabolites, or alteration of rumen pH (46). Therefore, the role of these bacteria in the rumen microbial community required further investigation to fully understand their impact on ruminant growth performance. Additionally, a significant positive correlation was observed between ruminal pH, propionate concentration, and the CH_4_/total gas emission ratio with the relative abundance of *Prevotella*, *Ruminococcus E*, *Acinetobacter*, and *RUG11690*. This might highlight the critical role these bacteria play in regulating rumen pH balance and promoting propionate production. Bacteria such as *Prevotella* and *Ruminococcus E* exhibit strong fermentative activity for propionate and may therefore be more active in lower pH environments (47). In contrast, the relative abundance of *Advenella* and *Bact 11* negatively correlated with pH, suggesting that these bacteria may dominate in promoting acetate production and help maintain a higher pH environment (48). The concentration of NH_3_-N was positively correlated with the relative abundance of *Succiniclasticum*, *Ruminococcus E*, and *Prevotella*, while negatively correlated with *Acinetobacter*. This suggested that *Succiniclasticum* and *Ruminococcus E* might play significant roles in nitrogen source utilization and the regulation of NH_3_-N concentration (49). *Ruminococcus E*, which was involved in both fiber degradation and nitrogen metabolism, might influence the overall nitrogen cycle by regulating ammonia nitrogen production and absorption (50). Additionally, the negative correlation between *Acinetobacter* abundance and NH_3_-N concentration suggested its potential role in protein degradation pathways and in regulating the rumen nitrogen balance by promoting NH_3_-N excretion (51).

## 5 Conclusion

With the increase of whole-plant quinoa silage supplemental level, the digestibility of dry matter, acetate concentration and cumulative CH_4_ emissions was enhanced, while the CH_4_ fraction of total gas emissions was decreased. The whole plant quinoa silage also increased the microbial function such as energy metabolism, amino acid metabolism and biosynthesis of secondary metabolites. Managing the inclusion rate of quinoa in practical production could serve as an effective strategy to optimize ruminant feed and mitigate greenhouse gas emissions. However, quinoa’s high fiber content may present challenges in providing adequate protein and energy. Consequently, when incorporating quinoa as a feed ingredient, it is essential to consider its effects on nutritional value, fermentation processes, and gas emissions comprehensively.

## Data Availability

The raw sequencing data have been deposited in the NCBI BioProject database with the accession number PRJNA1213034.

## Supplementary material

SUPPLEMENTARY TABLE S1 Differences in rumen bacterial phyla (Top 5) of silage prepared with alfalfa, quinoa, and their mixture.

SUPPLEMENTARY TABLE S2 Differences in rumen bacterial genera (top 22) of silage prepared with alfalfa, quinoa, and their mixture.
