# Supplementary material for: Effects of substituting alfalfa silage with whole plant quinoa silage on rumen fermentation characteristics and rumen microbial community of sheep in vitro
Source: Front Vet Sci. 2025 Apr 9;12:1565497. doi: 10.3389/fvets.2025.1565497 (PMC12016881; doi:10.3389/fvets.2025.1565497)
Supplement: SUPPLEMENTARY TABLE S1 — Differences in rumen bacterial phyla (Top 5) of silage prepared with alfalfa, quinoa, and their mixture. [file Table_1.DOCX]

**Table S1**. Differences in rumen bacterial phyla (top 5) of silage prepared with alfalfa, quinoa and their mixture

| Item | Treatment^1^ | | | | | Significance^3^ | | | |
| --- | --- | --- | --- | --- | --- | --- | --- | --- | --- |
|  | Q0 | Q30 | Q50 | Q70 | Q100 | SEM^2^ | T | L | Q |
| Bacteroidota | 57.24^a^ | 54.77^b^ | 53.62^b^ | 58.75^a^ | 48.88^c^ | 0.9449 | <.0001 | 0.053 | 0.081 |
| Firmicutes | 16.22^ab^ | 15.75^ab^ | 16.65^a^ | 15.34^b^ | 16.10^ab^ | 0.1685 | 0.1198 | 0.601 | 0.865 |
| Proteobacteria | 11.33^b^ | 5.77^d^ | 5.51^d^ | 6.32^c^ | 12.86^a^ | 0.8313 | <.0001 | 0.559 | <.0001 |
| Spirochaetota | 4.36^a^ | 1.88^b^ | 1.92^b^ | 1.85^b^ | 2.04^b^ | 0.2636 | <.0001 | 0.006 | <.0001 |
| Verrucomicrobiota | 7.70^c^ | 17.51^ab^ | 18.20^a^ | 14.17^b^ | 16.13^ab^ | 1.0918 | 0.0003 | 0.078 | 0.006 |
| ^1^ Alfalfa and quinoa were combined in proportions of 1:0 (Q0), 0.70:0.30 (Q30), 0.5:0.5 (Q50), 0.30:0.70 (Q70) and 0:1 (Q100).  ^2^ SEM, standard error of the mean. ^a-e^ means with different superscript letters in the same row differ (*P* < 0.05).  ^3^ T, treatment; L, linear; Q, quadratic. | | | | | | | | | |

**Table S2**. Differences in rumen bacterial genera (top 22) of silage prepared with alfalfa, quinoa and their mixture

| Item | Treatment^1^ | | | | | Significance^3^ | | | |
| --- | --- | --- | --- | --- | --- | --- | --- | --- | --- |
|  | Q0 | Q30 | Q50 | Q70 | Q100 | SEM^2^ | T | L | Q |
| *CAG-873* | 20.63^a^ | 16.95^b^ | 16.00^b^ | 20.95^a^ | 12.83^c^ | 0.8333 | <.0001 | 0.044 | 0.128 |
| *Prevotella* | 13.28^a^ | 10.97^b^ | 10.17^cd^ | 10.62^bc^ | 9.53^d^ | 0.3494 | <.0001 | <.0001 | <.0001 |
| *UBA1067* | 4.17^c^ | 11.28^a^ | 11.21^a^ | 8.74^b^ | 10.39^ab^ | 0.7522 | <.0001 | 0.059 | 0.007 |
| *Cryptobacteroides* | 7.66^a^ | 5.36^c^ | 5.41^c^ | 6.40^b^ | 6.24^b^ | 0.2285 | <.0001 | 0.282 | 0.003 |
| *Bact-11* | 4.11^d^ | 5.14^c^ | 5.11^c^ | 5.72^b^ | 6.84^a^ | 0.2464 | <.0001 | <.0001 | <.0001 |
| *Acinetobacter* | 6.69^a^ | 2.94^bc^ | 3.06^b^ | 2.61^c^ | 3.08^b^ | 0.4069 | <.0001 | 0.004 | <.0001 |
| *Limimorpha* | 2.18^c^ | 3.10^b^ | 3.97^a^ | 4.37^a^ | 3.40^b^ | 0.2121 | <.0001 | 0.007 | <.0001 |
| *SFMI01* | 2.78^b^ | 2.24^d^ | 2.12^d^ | 2.56^c^ | 3.13^a^ | 0.1005 | <.0001 | 0.158 | <.0001 |
| *F23-D06* | 0.69^c^ | 3.17^a^ | 2.76^b^ | 3.13^a^ | 2.99^a^ | 0.2526 | <.0001 | 0.005 | <.0001 |
| *Treponema_D* | 4.11^a^ | 1.67^b^ | 1.61^b^ | 1.56^b^ | 1.72^b^ | 0.2670 | <.0001 | 0.004 | <.0001 |
| *UBA1732* | 1.20^b^ | 2.05^a^ | 2.30^a^ | 1.90^a^ | 1.93^a^ | 0.1192 | 0.0148 | 0.120 | 0.008 |
| *Bacteroidaceae* | 1.65^c^ | 2.37^a^ | 2.12^b^ | 1.69^c^ | 1.50^d^ | 0.0880 | <.0001 | 0.118 | 0.001 |
| *Succiniclasticum* | 1.48^c^ | 1.57^c^ | 1.85^b^ | 2.13^a^ | 1.46^c^ | 0.0752 | 0.0007 | 0.353 | 0.020 |
| *Advenella* | 0.08^e^ | 0.36^d^ | 0.52^c^ | 1.17^b^ | 5.98^a^ | 0.5901 | <.0001 | <.0001 | <.0001 |
| *Paraprevotella* | 1.06^bc^ | 2.21^a^ | 2.26^a^ | 1.31^b^ | 0.96^c^ | 0.1531 | <.0001 | 0.331 | <.0001 |
| *RUG11690* | 2.91^a^ | 1.50^b^ | 1.27^c^ | 1.03^d^ | 0.87^d^ | 0.1965 | <.0001 | <.0001 | <.0001 |
| *Limivicinus* | 0.70^b^ | 1.11^a^ | 1.14^a^ | 0.82^b^ | 0.49^c^ | 0.0684 | <.0001 | 0.153 | <.0001 |
| *UBA11452* | 0.39^c^ | 0.99^a^ | 1.05^a^ | 0.67^b^ | 0.69^b^ | 0.0682 | <.0001 | 0.572 | 0.005 |
| *Succinivibrio* | 0.96^a^ | 0.49^c^ | 0.41^c^ | 0.67^b^ | 1.01^a^ | 0.0673 | <.0001 | 0.576 | <.0001 |
| *Faecousia* | 0.56^b^ | 0.64^ab^ | 0.71^ab^ | 0.76^a^ | 0.66^ab^ | 0.0253 | 0.1223 | 0.075 | 0.030 |
| unclassified_*Bacteria* | 0.31^c^ | 0.64^b^ | 0.83^a^ | 0.81^a^ | 0.67^b^ | 0.0510 | <.0001 | 0.007 | <.0001 |
| *Ruminococcus_E* | 0.80^a^ | 0.61^b^ | 0.72^ab^ | 0.69^ab^ | 0.45^c^ | 0.0342 | 0.0004 | 0.006 | 0.014 |
| ^1^ Alfalfa and quinoa were combined in proportions of 1:0 (Q0), 0.70:0.30 (Q30), 0.5:0.5 (Q50), 0.30:0.70 (Q70) and 0:1 (Q100).  ^2^ SEM, standard error of the mean. ^a-e^means with different superscript letters in the same row differ (*P* < 0.05).  ^3^ T, treatment; L, linear; Q, quadratic. | | | | | | | | | |
